# Correction: Mechanistic study on NO reduction by sludge reburning in a pilot scale cement precalciner with different CO_2_ concentrations

**DOI:** 10.1039/d0ra90006k

**Published:** 2020-01-15

**Authors:** Xiang Xiao, Ping Fang, Jian-Hang Huang, Zi-Jun Tang, Xiong-Bo Chen, Hai-Wen Wu, Chao-Ping Cen, Zhi-Xiong Tang

**Affiliations:** South China Institute of Environmental Sciences, Ministry of Ecology and Environment Guangzhou 510655 Guangdong China fangping@scies.org; The Key Laboratory of Water and Air Pollution Control of Guangdong Province Guangzhou 510655 Guangdong China

## Abstract

Correction for ‘Mechanistic study on NO reduction by sludge reburning in a pilot scale cement precalciner with different CO_2_ concentrations’ by Xiang Xiao *et al.*, *RSC Adv.*, 2019, **9**, 22863–22874.

Affiliation a was incorrect in the published article; the correct version is shown here.

The Royal Society of Chemistry apologises for these errors and any consequent inconvenience to authors and readers.

## Supplementary Material

